# Accumulation of ibuprofen in endemic amphipods of Lake Baikal

**DOI:** 10.7717/peerj.21008

**Published:** 2026-04-09

**Authors:** Tamara Yu Telnova, Maria M. Morgunova, Sophie S. Shashkina, Maria E. Dmitrieva, Victoria N. Shelkovnikova, Olga E. Lipatova, Ekaterina V. Malygina, Natalia A. Imidoeva, Alexander Yu Belyshenko, Tatiana N. Vavilina, Arcadii N. Matveev, Evgenia A. Misharina, Denis V. Axenov-Gribanov

**Affiliations:** 1 Bioorganics Research and Educational Center, Irkutsk State University, Irkutsk, Russia; 2 UNESCO Chair on Water Resources, Irkutsk State University, Irkutsk, Russia; 3 Institute of Biological Sciences, Irkutsk State University, Irkutsk, Russia

**Keywords:** Amphipods, Pharmaceutical pollution, Ibuprofen, Lake baikal, Accumulation, Endemics

## Abstract

Pharmaceutical pollutants, including ibuprofen, are now ubiquitously detected in global aquatic ecosystems, exerting significant negative ecological impacts. Lake Baikal organisms have been documented to accumulate ibuprofen and related contaminants. This study quantified ibuprofen concentrations within Lake Baikal’s endemic amphipod fauna using high-performance liquid chromatography coupled with mass spectrometry. We analyzed specimens representing key ecological groups across the genera *Eulimnogammarus*, *Brandtia*, *Ommatogammarus*, and *Pallasea*. Ibuprofen concentrations ranged from 4.19 ng/g to 1,151.32 ng/g (wet weight), confirming consistent contamination in both littoral and deep-water endemic amphipod populations. Crucially, our data provide the first evidence suggesting amphipods, or their associated symbiotic microbiota, may metabolize ibuprofen. Interspecific accumulation patterns were identified, with *Eulimnogammarus* sp. and *Brandtia* sp. exhibiting distinct profiles. Furthermore, accumulation was significantly higher during spring compared to autumn samples. A negative correlation emerged between ibuprofen concentration and amphipod body mass within species. Several populations contained non-detectable levels. These findings demonstrate that endemic amphipods within Lake Baikal’s natural environment are exposed to and bioaccumulate the pharmaceutical pollutant ibuprofen, exhibiting species-specific, seasonal, and allometric variation in uptake.

## Introduction

Pharmaceuticals are indispensable in modern healthcare and daily life, safeguarding public health while enhancing quality and longevity. Non-steroidal anti-inflammatory drugs (NSAIDs) constitute a major therapeutic class, extensively employed in human and veterinary medicine for their analgesic, antipyretic, and anti-inflammatory properties (Parolini, 2020). Portions of this text were previously published as part of a preprint (Telnova et al., 2025).

Global accessibility and prevalence drive escalating NSAID demand and production. However, regulatory oversight remains limited, with most NSAIDs available over-the-counter (Jan-Roblero & Cruz-Maya, 2023). An estimated 30 million individuals use NSAIDs daily, exceeding 300 million users annually worldwide (Bindu, Mazumder & Bandyopadhyay, 2018). This high consumption poses significant environmental challenges (Nieto et al., 2017; Jurado, Vázquez-Suñé & Pujades, 2021) due to incomplete drug metabolism in organisms, inadequate disposal practices and insufficient removal by wastewater treatment facilities. Following ingestion, a substantial fraction of NSAIDs is excreted unmetabolized or as bioactive compounds (Marchlewicz, Guzik & Wojcieszyńska, 2015), ultimately entering aquatic ecosystems. The resultant influx of pharmaceutical pollutants exerts detrimental effects on freshwater biota. Monitoring studies consistently detect NSAIDs—including acetylsalicylic acid, paracetamol (acetaminophen), diclofenac, ketoprofen, ibuprofen, and naproxen—in freshwater systems globally (Tyumina et al., 2020), underscoring their pervasive environmental presence.

The consequences of the ecotoxicological impact of NSAIDs on aquatic organisms include reproductive and endocrine disorders in animals, early development of organisms, and immune responses (Zhao et al., 2024). For example, research by Xu et al. (2019) has shown that naproxen is acutely toxic to aquatic organisms and thus causes chronic effects. It has also been described that NSAIDs are capable of forming free radicals that cause oxidative stress. This process can exacerbate cell inflammation, cause apoptosis and necrosis, damage DNA bases, and cause mitochondrial dysfunction (Kıran, Otlu & Karabulut, 2023).

Notably, ibuprofen stands out among NSAIDs due to its extensive clinical use and inclusion on the World Health Organization’s List of Essential Medicines (Chopra & Kumar, 2020). However, its high global consumption—with annual production exceeding 300,000 tonnes (Marchlewicz, Guzik & Wojcieszyńska, 2015)—presents a significant environmental risk. Ibuprofen exhibits limited aqueous solubility and undergoes incomplete metabolism in humans. Following excretion, it enters the environment either unmetabolized or as biotransformation products, primarily hydroxyibuprofen, carboxyibuprofen, carboxyhydratropic acid (Chopra & Kumar, 2020), and 4-isobutylcatechol (Larsen et al., 2019). These metabolites often undergo further transformation and are often more toxic to aquatic organisms than the parent compound (Chopra & Kumar, 2020).

Global monitoring studies confirm ibuprofen’s pervasive presence in aquatic systems. Reported concentrations in surface waters range from 0.98 to 414 ng/L across diverse regions including Canada, France, China, Greece, Korea, Taiwan, and Uganda (Kim et al., 2009; Vulliet, Cren-Olivé & Grenier-Loustalot, 2011; Almeida et al., 2013; Luo et al., 2014; Nantaba et al., 2020). Conversely, groundwater studies in Europe report lower but still detectable levels, ranging from 3 to 395 ng/L (Luo et al., 2014), underscoring its environmental persistence.

Furthermore, ibuprofen and its metabolites exert demonstrable toxicity on aquatic organisms (Das et al., 2019). Field studies confirm bioaccumulation across taxa: *Hydropsyche* spp. caddisflies in Spain’s Segre River contained 184 ng/g ibuprofen (Huerta et al., 2015), while *Gammarus fossarum* amphipods downstream of a wastewater treatment plant in southern France showed concentrations of 60.6–105.4 ng/g (Berlioz-Barbier et al., 2014). In urbanized Chinese rivers, ibuprofen was detected in phytoplankton (14.5–35.8 ng/g), zooplankton (20.9–48.9 ng/g), and benthic invertebrates (freshwater shrimp, mussels, snails: 4.8–11.6 ng/g) (Yang et al., 2020). Laboratory studies indicate adverse effects occur at concentrations typically orders of magnitude higher than environmental levels (10–100 mg/L) (Pajić et al., 2023).

Ibuprofen at a concentration of 250 ng/l has been described to cause endocrine disorders in *Mytilus galloprovincialis*. Also, the presence of the drug can induce damage to the membranes of the digestive glands in mussels and an increase in the level of lipid peroxidation. *Danio* fish exhibited changes in antioxidant protection after exposure to ibuprofen at concentrations of 0.1–11 µg/L (Falfushynska et al., 2022). *Danio rerio* also exhibited head malformations, skeletal deformities, hypopigmentation, pericardial edema, and cardiac arrhythmia (Sanchez-Aceves et al., 2021).

Studies by Han et al. (2010) *in vitro* showed that ibuprofen increases the production of 17-β-oestradiol and reduces testosterone production and aromatase activity in test subjects such as *Oryzias latips*, *Moina macrocopa*, and *Daphnia magna* (Han et al., 2010). Ibuprofen at a concentration of 10 mg/l had an inhibitory effect on the maturation of the ovaries of adult female crabs, *Neohelice granulate* (Lofrano et al., 2022).

Documented toxicity spans diverse aquatic taxa, including echinoderms (*Asterias rubens*, *Psammechinus miliaris*), polychaetes (*Arenicola marina*), microalgae (*Navicula* sp., *Chlorella vulgaris*, *Acutodesmus obliquus*, *Chlamydomonas reinhardtii*, *Nannochloropsis limnetica*), and crustaceans (*Daphnia magna*) (Geiger, Hornek-Gausterer & Saçan, 2016; Grzesiuk, Wacker & Spijkerman, 2016; Du et al., 2016; Zanuri, Bentley & Caldwell, 2017; Ding, Li & Li, 2019).

These findings underscore that ibuprofen contamination poses a potential yet understudied threat to ancient aquatic ecosystems. Lake Baikal—a tectonic-formed UNESCO World Heritage site in southeastern Siberia—represents a critical case study as Earth’s deepest lake (Moore et al., 2009; Moore et al., 2019; Rusinek et al., 2012) and one of its most ancient, with recent geological evidence indicating an age exceeding 60 million years (Matz & Efimova, 2017). This evolutionary cradle harbors >2,600 animal species exhibiting exceptional endemism (Timoshkin et al., 2016), heightening vulnerability to anthropogenic pollutants like ibuprofen.

As in all aquatic ecosystems, pollutants in Lake Baikal undergo trophic transfer *via* nekton, plankton, and benthos (Rusinek et al., 2012). Among endemic benthic taxa, amphipods (Amphipoda, Crustacea) represent a hyperdiverse and ubiquitous group occupying all depth zones and substrate types (Takhteev, 2019). This ecological dominance positions them as sentinel organisms for early pollutant exposure.

In Lake Baikal, as in other aquatic ecosystems, ibuprofen, along with other pollutants, enters the water through atmospheric and domestic wastewater, as well as through the misuse and improper disposal of medicines. It is noteworthy that pharmaceutical pollution of Lake Baikal is largely due to the lack of treatment facilities near populated areas, and the discharge of industrial wastewater is generally prohibited under Federal Law No. 94-FZ “On the Protection of Lake Baikal”.

Critically, recent research confirms Baikal amphipods bioaccumulate multiple pharmaceuticals—including acetylsalicylic acid, paracetamol, tetracycline antibiotics, and notably ibuprofen (Telnova et al., 2024). The persistent detection of ibuprofen, a compound with established ecotoxicity, signals direct risks to endemic species and broader lake ecosystem integrity. To address the main knowledge gaps, this study quantifies ibuprofen concentrations in Baikal-endemic amphipods for specific species and populations for the first time and evaluates the dynamics of seasonal accumulation.

## Materials & Methods

The current study focused on adult amphipods spanning key ecological niches within Lake Baikal. Specimens included littoral species (*Eulimnogammarus cyaneus* (Dybowsky, 1874), *E. verrucosus* (Gerstfeldt, 1858), and unidentified *Eulimnogammarus* specimens), free-living sublittoral species (*Brandtia* sp. (Bate, 1862)), sublittoral benthic species (*Pallasea* sp. (Bate, 1862)), and deep-water species *Ommatogammarus flavus* (Dybowsky, 1874). Detailed ecological and physiological characterization of these taxa was well described in earlier studies (Axenov-Gribanov et al., 2016; Shirokova et al., 2025; Drozdova et al., 2025).

Amphipods related to species *E. verrucosus* were collected from littoral zones at: Kultuk, Listvyanka, Bolshoye Goloustnoye, Ust-Barguzin, and Buguldeika settlements (South and Middle Baikal) during spring 2023. Additional specimens were obtained from the Angara River in Irkutsk city. The place of sampling in Angara was located upstream of municipal wastewater treatment facilities. Listvyanka populations of *E. verrucosus* were sampled seasonally (spring and autumn 2023). The distance of the settlements from the coastline of Lake Baikal, where the samples were taken, was as follows: Kultuk settl. –180 m, Listvyanka settl. –450 m, Buguldeyka settl. –1.2 km, B. Goloustnoye settl. –954 m, Ust-Barguzin settl. –6.2 km. The distance between Irkutsk and the Angara River shoreline is 13 m.

Specimens of *E. cyaneus* were obtained from the littoral zone of Angara River during spring 2023. Amphipods of *Brandtia* sp. were collected concurrently at two sites: the Angara River and Listvyanka settlement. *O. flavus* was collected from the profundal zone (70 m depth) near Buguldeika settlement using hydrobiological traps (spring 2023). All specimens were collected with hydrobiological nets except where noted.

The maximum body size of amphipods reached 1.5 mm. The weight of amphipods varied: the minimum weight of 0.02 and 0.03 g was observed in amphipods of unidentified species of the genera *Eulimnogammarus* sp. and *Brandtia* sp. The maximum weight was recorded in *Eulimnogammarus verrucosus* at 0.71 g. The age of amphipods may be related to body weight.

**Figure 1:**
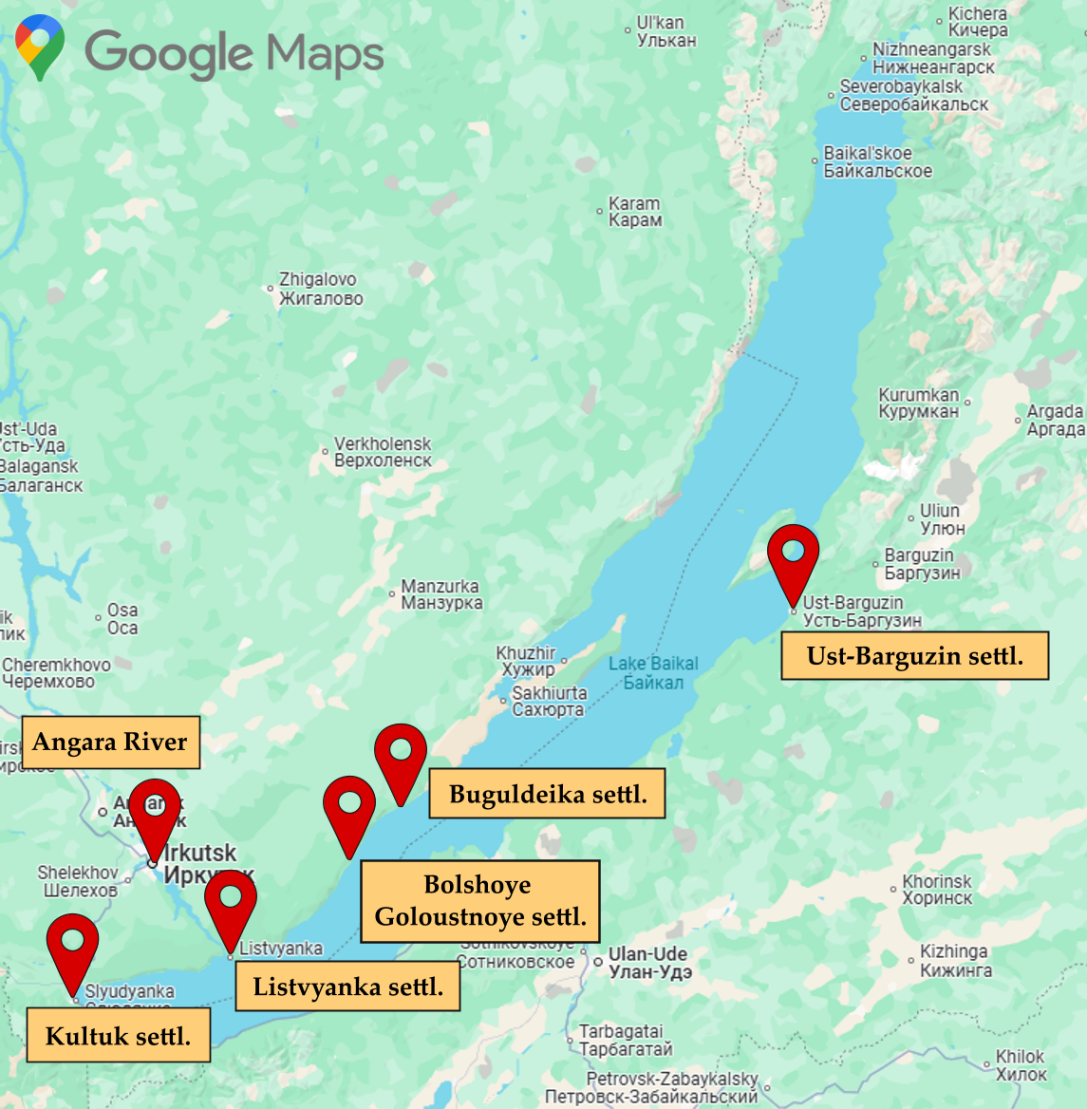
Map of amphipod sampling sites, Lake Baikal. The map of Lake Baikal shows sampling sites for endemic amphipods. The map fragment was taken from the free Google Maps mapping service. The use of a map fragment is allowed without the need to request permission, and the figure itself has a link to Google Maps.

All specimens were immediately transferred to two mL plastic Eppendorf tubes, flash-frozen in liquid nitrogen, and stored at −196 °C. Sampling locations are shown in Fig. 1. Lake Baikal experiences intense recreational pressure as a major tourism hub, with all sampling sites situated in highly frequented destinations (Aleksandrova et al., 2021). This anthropogenic context heightens contamination risks from pharmaceutical residues in nearshore ecosystems.

The study included two stages, and involved qualitative and quantitative assessments of ibuprofen content in amphipods. Each amphipod was weighed individually on an analytical balance (Sartorius CE224-C, St. Petersburg, Russia) and homogenized three times in a vibratory ball mill (BABRx1, Mycotech, Irkutsk, Russia) with acetonitrile (Sigma-Aldrich, St. Louis, MO, USA, 34851−2.5L) in a ratio of 1:10. Additional information on amphipod extraction is provided in the study by Telnova et al. (2024). After each grinding cycle, samples were briefly centrifuged at 1,000 rpm for 1 min (Armed LC-04B, St. Petersburg, Russia) to separate supernatant and debris. Supernatant was transferred into new 2-mL Eppendorf tubes, and debris was re-extracted. After complete extraction, samples were brought to a final volume of four mL with acetonitrile. The tubes were centrifuged (Armed LC-04B, St. Petersburg, Russia) for 10 min at 3,000 rpm. 2-mL aliquot of supernatant was transferred into glass vials and concentrated in a vacuum oven (Stegler VAC-52 FCD-3000, Shanghai, China). The dried residues were reconstituted in one mL of 40:60 (v/v) acetonitrile/milli-Q water. Extracts underwent ultrasonication (Granbo, GS0201) for 10 min and were transferred to microtubes (Eppendorf tubes). 50 µL of 10% trichloroacetic acid (LLC “LabTehKomplekt”, TU-6-09-1926-77) solution was added to each sample. Microtubes were vortex-mixed for 1 min and centrifuged at 16,000 rpm during 10 min (Microspin-12, Biosan Riga, Latvia) (Gonzalez-Rey & Bebianno, 2012). Prior to analysis, samples were filtered through 13-mm PVDF syringe membranes (0.45-µm pore size). An aliquot of 200 µl filtrate was transferred to vials for analysis using high-performance liquid chromatography coupled with mass spectrometry (HPLC-MS).

During the study, three types of samples were measured: amphipod extracts (a), ibuprofen analytical standard solution (Certified Reference Material 11559-2020, NCAS, Moscow, Russia) (b), and amphipod extracts spiked with ibuprofen standard (c). The analytical standard solution was used to optimize ionization parameters and evaluate chromatographic system performance (Eraga et al., 2015). Subsequently, amphipod extracts were analyzed both unmodified and following standard addition. The concentration of the ibuprofen analytical standard working solution was 72 ng/mL. Spiked samples consisted of 600 µL amphipod extract and 100 µL ibuprofen analytical standard solution (final volume: 700 µL) (Bashyal, 2018; Dowling et al., 2008). A linear calibration curve was generated for ibuprofen concentrations ranging from 0.025 ng/mL to 50 ng/mL. The Limit of Detection (LOD) is 30 pg/mL, and the Limit of Quantitation (LOQ) is 0.025 ng/mL.

Screening for ibuprofen in Baikal endemic amphipods was performed using an Agilent Infinity II (2019) LC-MS/MS system equipped with an Agilent 6470B triple quadrupole mass spectrometer. Chromatographic separation used an Agilent Poroshell C18 column (2.1  × 50 mm) maintained at 30 °C. Table 1 presents the program for HPLC separation of samples containing ibuprofen. Mass spectrometer setup program: ion source gas temperature: 300 °C; ion source gas flow: 5 L/min; nebuliser: 45 psi; drying gas temperature: 250 °C; drying gas flow: 11 L/min; capillary voltage: 3,500 V; sample volume: 1 µL. Ibuprofen detection used MRM transition 205.1 → 161.1 (Gost 32881-2014, 2016, Moscow, Russia).

The study involved analysis of 218 specimens of Baikal amphipods, including *E. verrucosus* (*n* = 180), *O. flavus* (*n* = 20), *E. cyaneus* (*n* = 3), and unidentified amphipods of the genera *Eulimnogammarus* (*n* = 8), *Brandtia* sp. (*n* = 6), and *Pallasea* sp. (*n* = 1). Samples of *E. verrucosus*, *Brandtia* sp. and *Pallasea* sp. comprised single individuals. Analyses of *O. flavus* and *E. cyaneus* utilized pooled samples containing two individuals each. There were 55 specimens of the amphipod *E. verrucosus* collected in the spring, while there were 120 specimens collected in the autumn.

The statistical processing was performed in Past software (V4.03) using the Kruskal–Wallis H test (one-way ANOVA on ranks as a non-parametric method) for the analysis of qualitative data. The Bonferroni sequential significance model was used. One-way ANOVA, *t*-test and F-test were used to analyze quantitative data. The effects of factors “year of sampling” and “wet weight of amphipods” were reported based on two-way ANOVA. To determine which group means were significantly different from each other, Tukey’s *post-hoc* tests were performed. Differences between the mean values of the parameters were considered significant at *p* ≤ 0.05.

## Results

### Qualitative assessment of the ibuprofen presence in Baikal endemic amphipod

**Figure 2:**
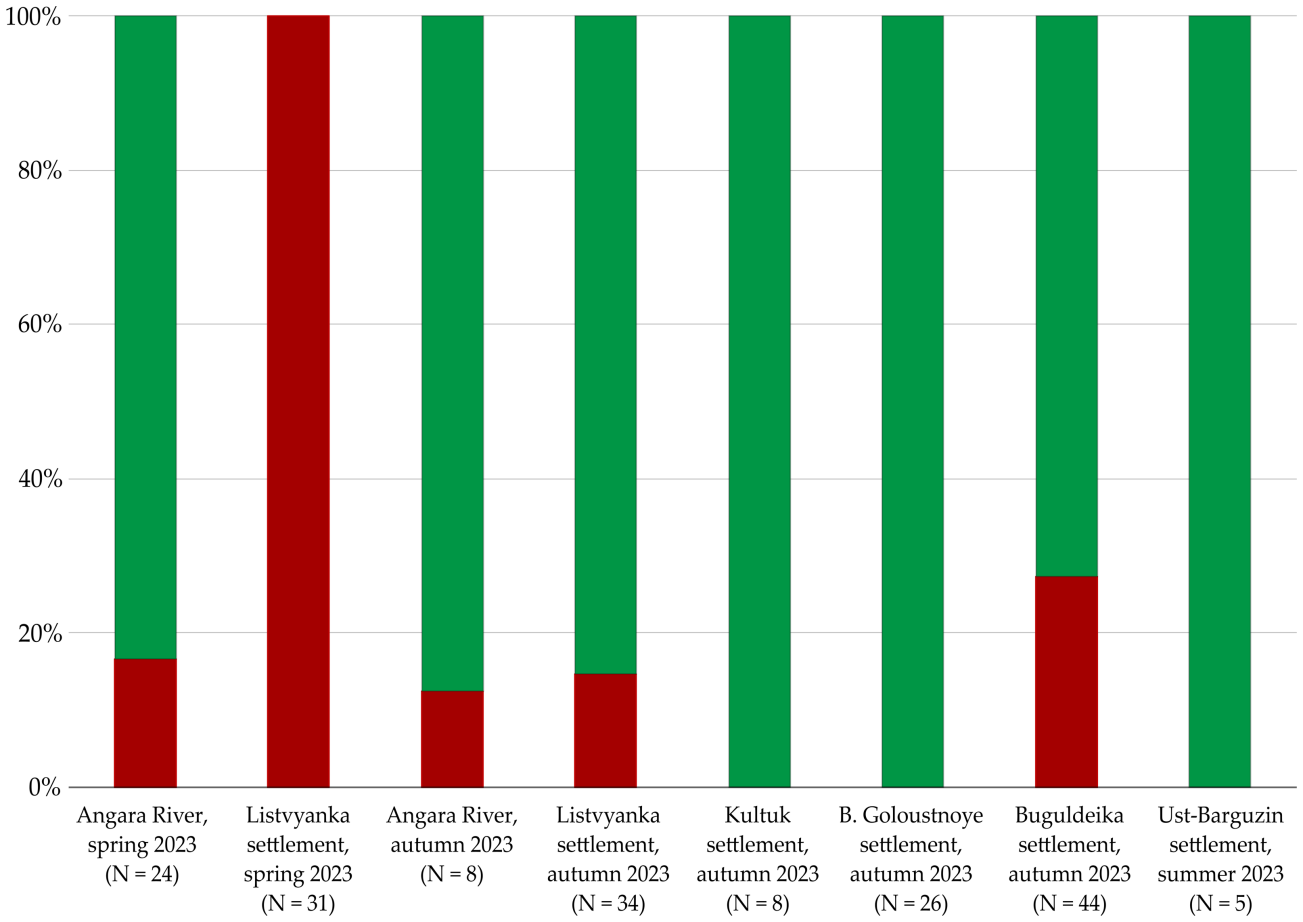
Qualitative assessment of ibuprofen in distinct populations of *E. verrucosus*. Red—*E. verrucosus* contaminated with ibuprofen; Green—clean samples of amphipods in which ibuprofen was not detected. Note: Contamination frequency is the percentage of analyzed amphipods in which the contaminant was detected.

A qualitative assessment of ibuprofen presence was conducted in amphipods of the genera *Eulimnogammarus* spp., collected in spring, summer, and autumn 2023; *Brandtia* spp. and *Pallasea* spp., collected in spring 2023; and *Ommatogammarus* spp., collected in spring 2023. The qualitative analysis demonstrated that ibuprofen was detected in *E. verrucosus* collected in the Angara River, Listvyanka settl., and Buguldeyka settl. The frequency of ibuprofen contamination detected in amphipods sampled in spring ranged from 18 to 100%. At the same time, the frequency of ibuprofen contamination detected in amphipods sampled in autumn ranged from 12 to 27%. Ibuprofen was not detected in amphipods collected in Bolshoye Goloustnoye, Kultuk, and Ust-Barguzin settlements (Fig. 2).

**Table 1:** HPLC gradient program for ibuprofen separation. The table shows the mobile phase gradient program.

Time, min	Solution B100% Acetonitrile, %	Flow Rate, mL/min
1.0	40	0.4
4.0	98	0.4
6.0	98	0.4
7.0	40	0.4
7.5	40	0.4

The chromatogram of the analytical standard ibuprofen, the chromatogram of ibuprofen detected in amphipods, and the chromatogram of the amphipod extract added to the ibuprofen standard show identical retention times and mass spectra, confirming the presence of ibuprofen in endemic amphipods. The retention time is 3.4 min.

Ibuprofen was detected in the deep-water amphipod *O. flavus* collected at 70 m depth near Buguldeika settl. One of 20 analyzed specimens tested positive for ibuprofen. Also, qualitative assessment confirmed ibuprofen presence in *E. cyaneus* and *Pallasea* sp. However, insufficient biomaterial precluded quantification of contamination levels or internal ibuprofen concentrations.

### Quantitative assessment of ibuprofen content in Baikal endemic amphipod

**Figure 3:**
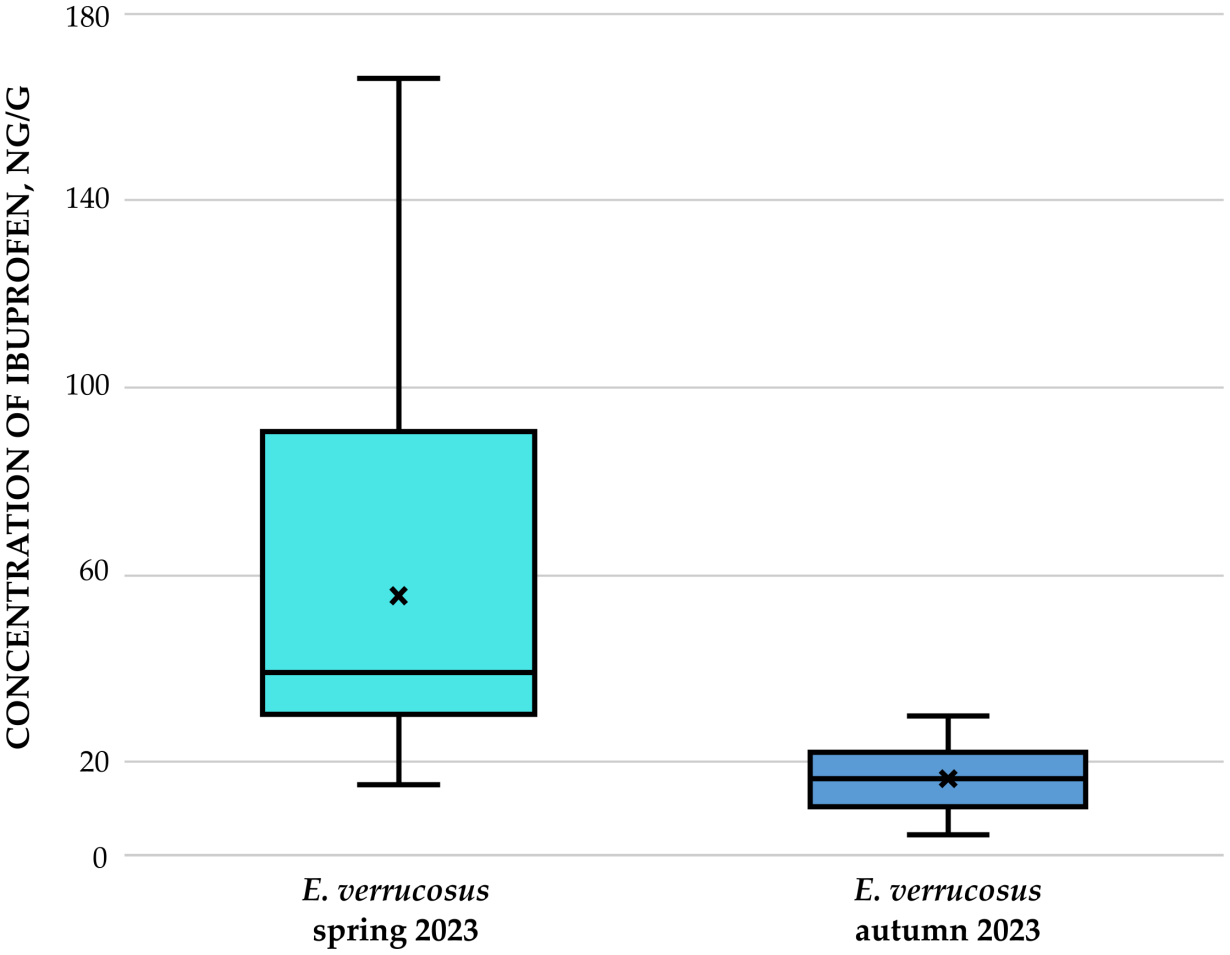
Concentration of ibuprofen (in ng/g) in amphipods of species *E. verrucosus* collected in two seasons in Listvyanka settl. in 2023. The seasonal difference between ibuprofen concentrations in *E. verrucosus* amphipods sampled in Listvyanka settlement is shown.

Quantitative assessment of ibuprofen in *E. verrucosus* from Listvyanka settl. revealed significantly higher accumulation during spring 2023 compared to autumn 2023 (*p* = 0.001). Spring amphipods accumulated twice the ibuprofen levels measured in their autumn counterparts (Fig. 3). The reason for seasonal differences may be related either to varying exposure concentrations or, possibly, to varying physiological characteristics of amphipods depending on the time of year. Varying exposure concentrations may be related to seasonal diseases.

In *E. verrucosus* collected during spring, ibuprofen concentrations ranged from 14.92 to 166.38 ng/g, while wet weights ranged from 0.074 to 0.77 g. During autumn, concentrations ranged from 4.19 to 29.65 ng/g, with wet weights spanning 0.21 to 0.43 g.

**Figure 4:**
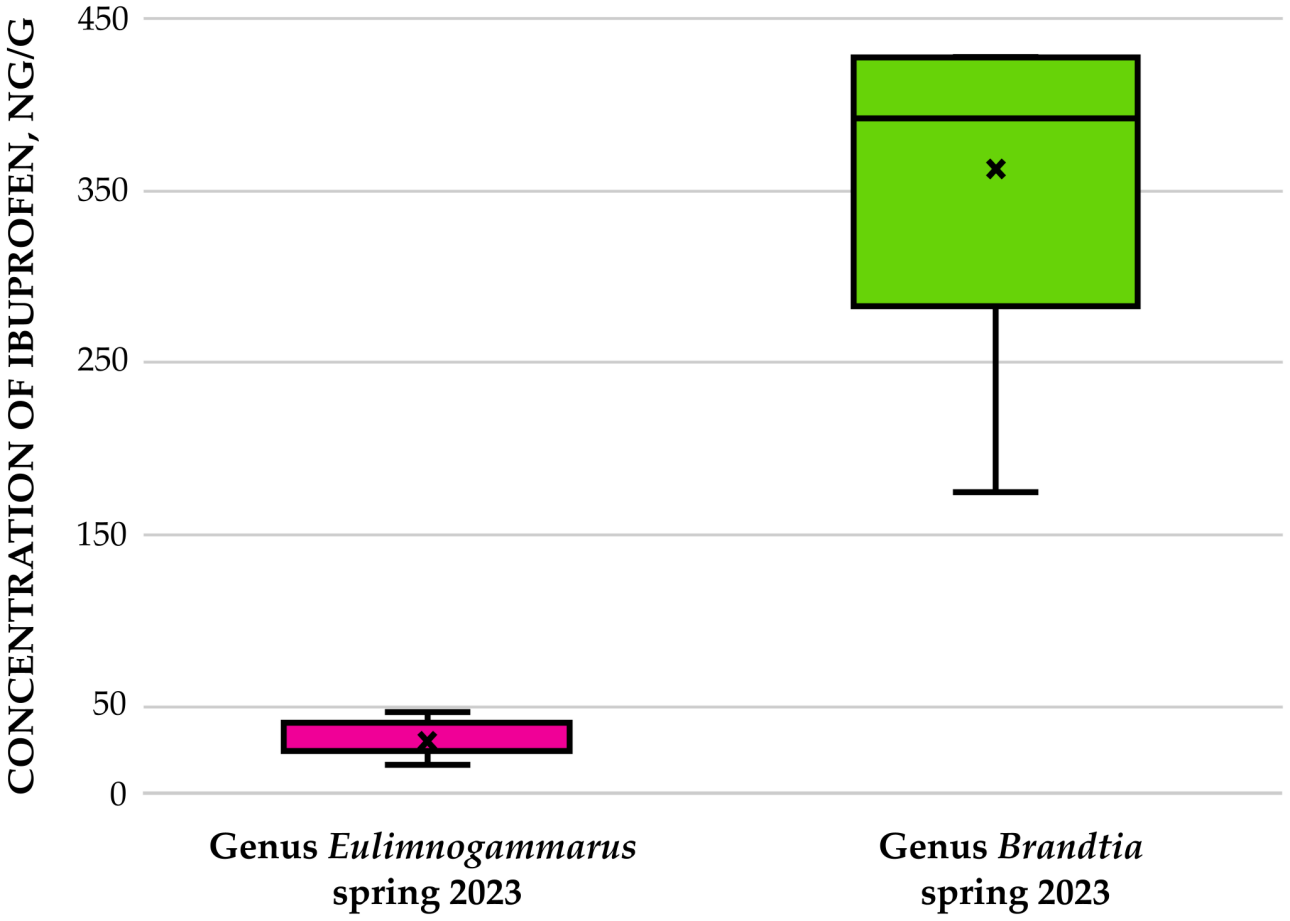
Concentration of ibuprofen (in ng/g) in amphipods of genera *Eulimnogammarus* and *Brandtia*, collected in the Angara River. Interspecific differences in the accumulation of ibuprofen in amphipods are shown.

Amphipods of the genera *Eulimnogammarus* and *Brandtia* collected from the Angara River in spring 2023 were subsequently analyzed. Quantitative assessment revealed significant interspecific differences in ibuprofen accumulation: *Brandtia* specimens accumulated fourfold higher concentrations than *Eulimnogammarus* (*p* = 0.001; Fig. 4). In amphipods of the genus *Eulimnogammarus*, ibuprofen concentrations ranged from 16.84 to 46.50 ng/g, with individual wet weights spanning 0.28 g to 0.44 g. For amphipods of the genus *Brandtia*, wet weights ranged from 0.028 g to 0.068 g, while ibuprofen concentrations ranged from 174.9 to 439.69 ng/g.

**Figure 5:**
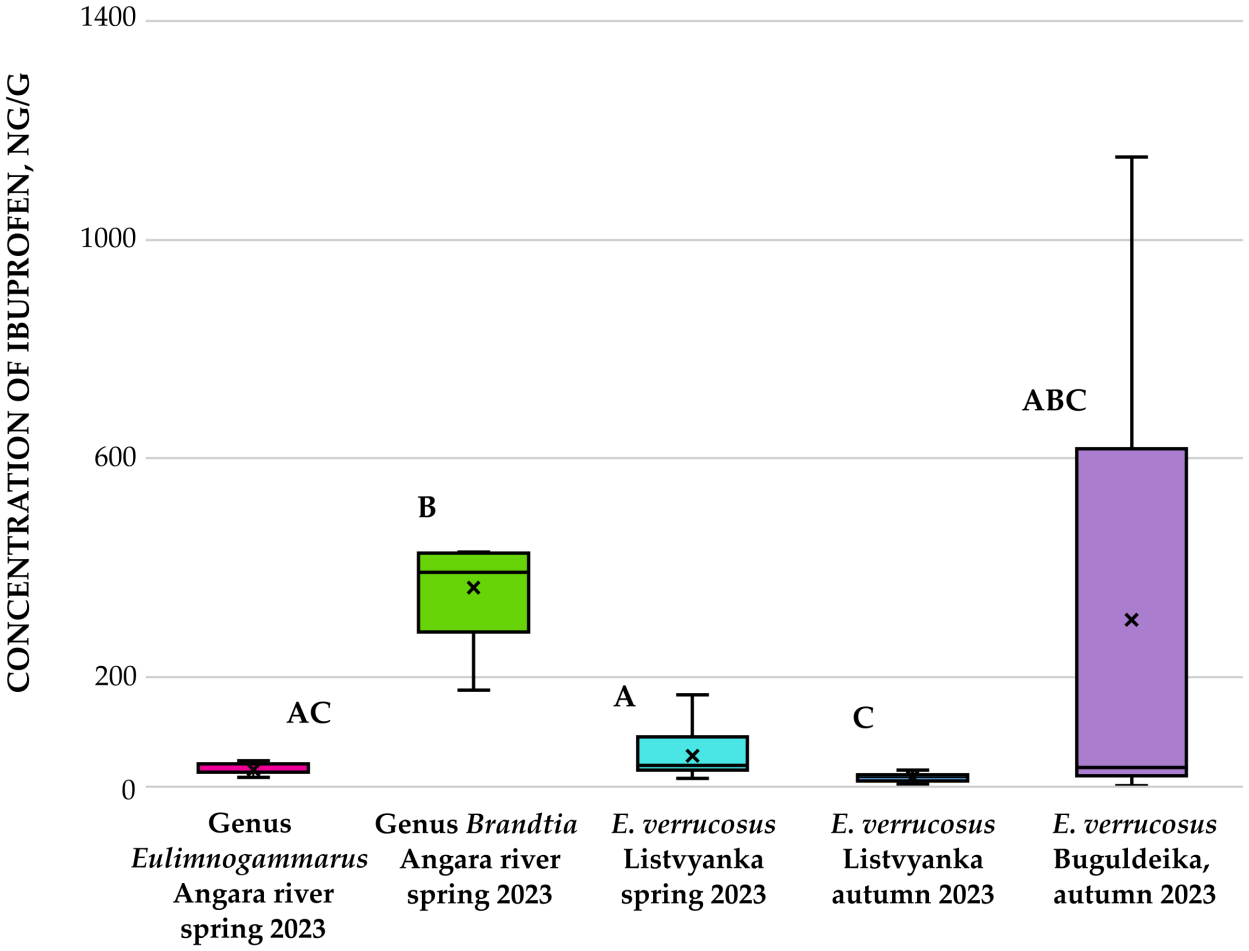
Concentration of ibuprofen (in ng/g) in amphipods collected in two seasons in 2023. Note: letter designations of amphipod groups denote statistically significant differences.

The maximum ibuprofen concentration was detected in *E. verrucosus* collected during autumn near Buguldeika settl. (Fig. 5). For specimens with wet weights ranging from 0.105 g to 0.307 g, ibuprofen levels ranged from 18.33 to 1,151.32 ng/g. In the studied individual of *Pallasea* sp. (wet weight: 0.130 g), ibuprofen was detected at 84.86 ng/g, while the concentration in deep-water *O. flavus* (wet weight: 0.39 g) was 5.15 ng/g.

**Figure 6:**
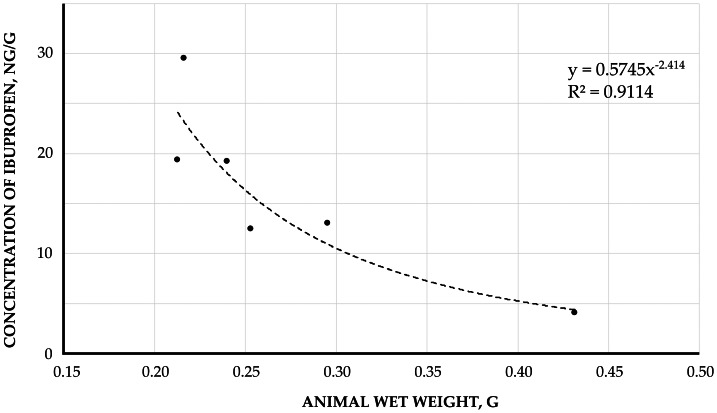
The correlation between the concentration of accumulated ibuprofen and wet weight of amphipod *E. verrucosus* (in ng/g). Samples were collected in Listvyanka settlement in autumn 2023.

**Figure 7:**
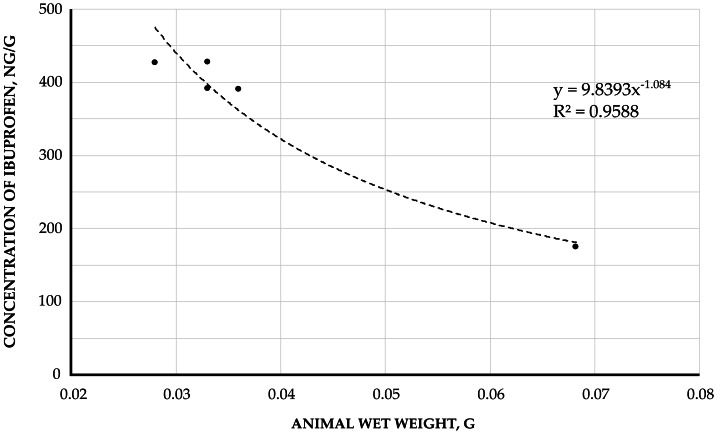
The correlation between the concentration of accumulated ibuprofen and wet weight of amphipod *Brandtia* (in ng/g). Samples appear in the Angara River, which flows within the city of Irkutsk. Sampling time: spring 2023.

Furthermore, a dependence of ibuprofen concentration on organism wet weight was observed in *E. verrucosus* collected during autumn near Listvyanka settl. (Fig. 6). In amphipods with wet weights from 0.21 to 0.29 g, the maximum ibuprofen concentration reached 29.65 ng/g, while specimens weighing 0.43 g contained ibuprofen at 4.19 ng/g. This weight-concentration relationship was also evident in *Brandtia* spp. from the Angara River (Fig. 7), where amphipods weighing 0.028−0.036 g showed concentrations of 385.03–431.63 ng/g, whereas a 0.068 g specimen accumulated ibuprofen at 174.90 ng/g.

**Figure 8:**
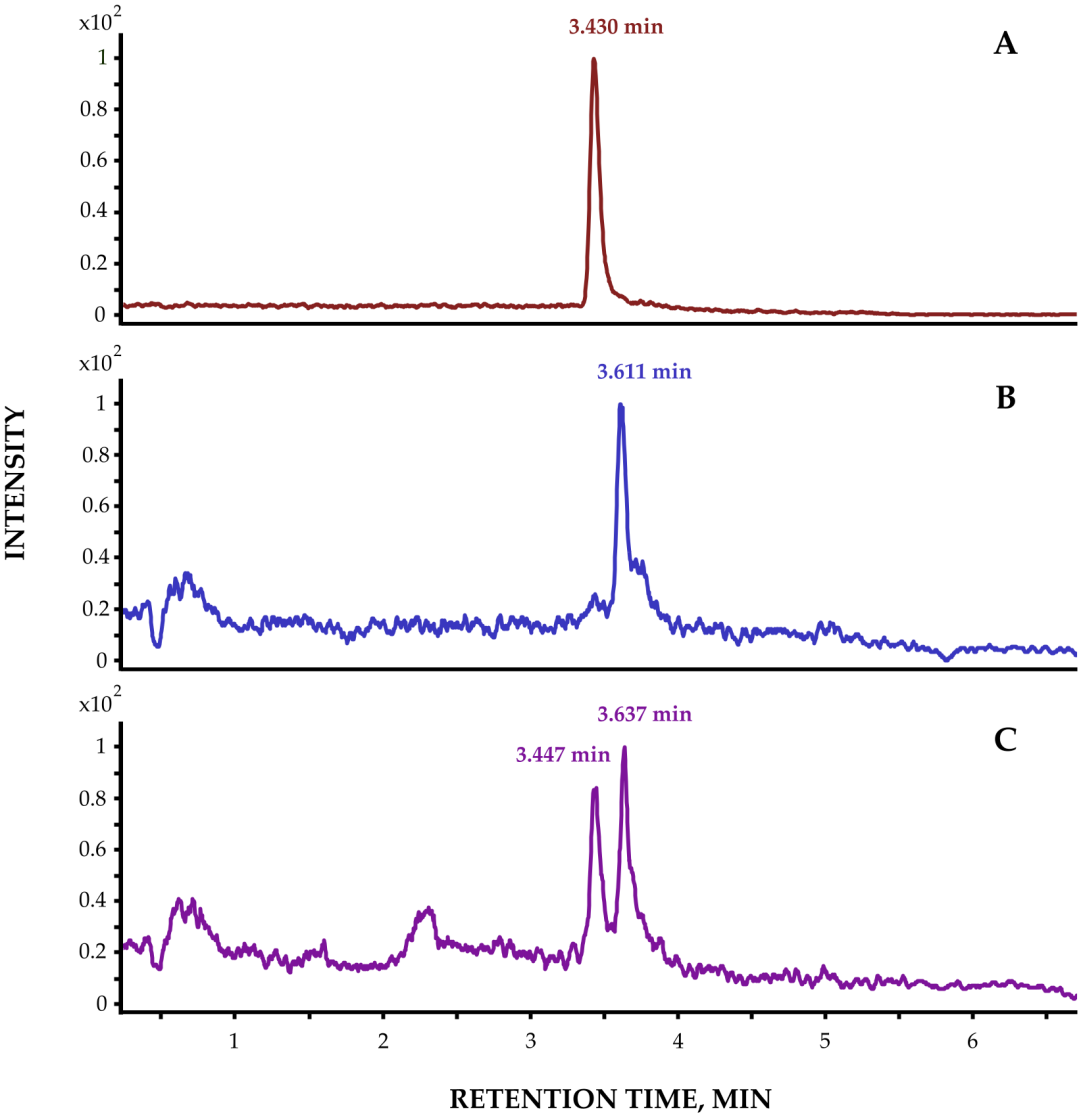
Representative chromatograms. (A) Ibuprofen analytical standard; (B) Suspected ibuprofen metabolite in *E. verrucosus* extract; (C) *E. verrucosus* extract spiked with ibuprofen standard.

**Figure 9:**
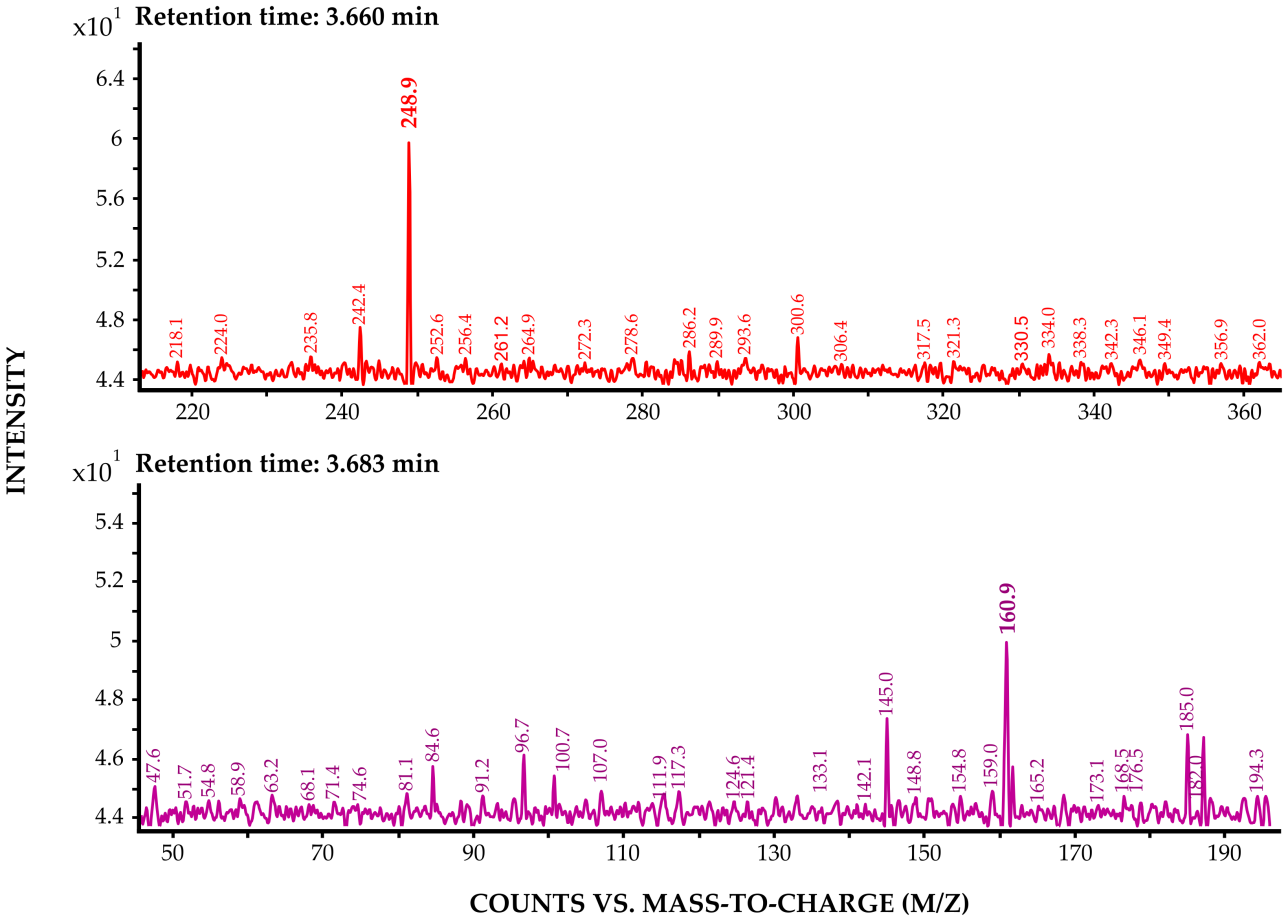
Spectra of the precursor ion (A) and product ion (B) of ibuprofen detected in the samples. The spectra of the precursor ion and the ion product of ibuprofen.

**Figure 10:**
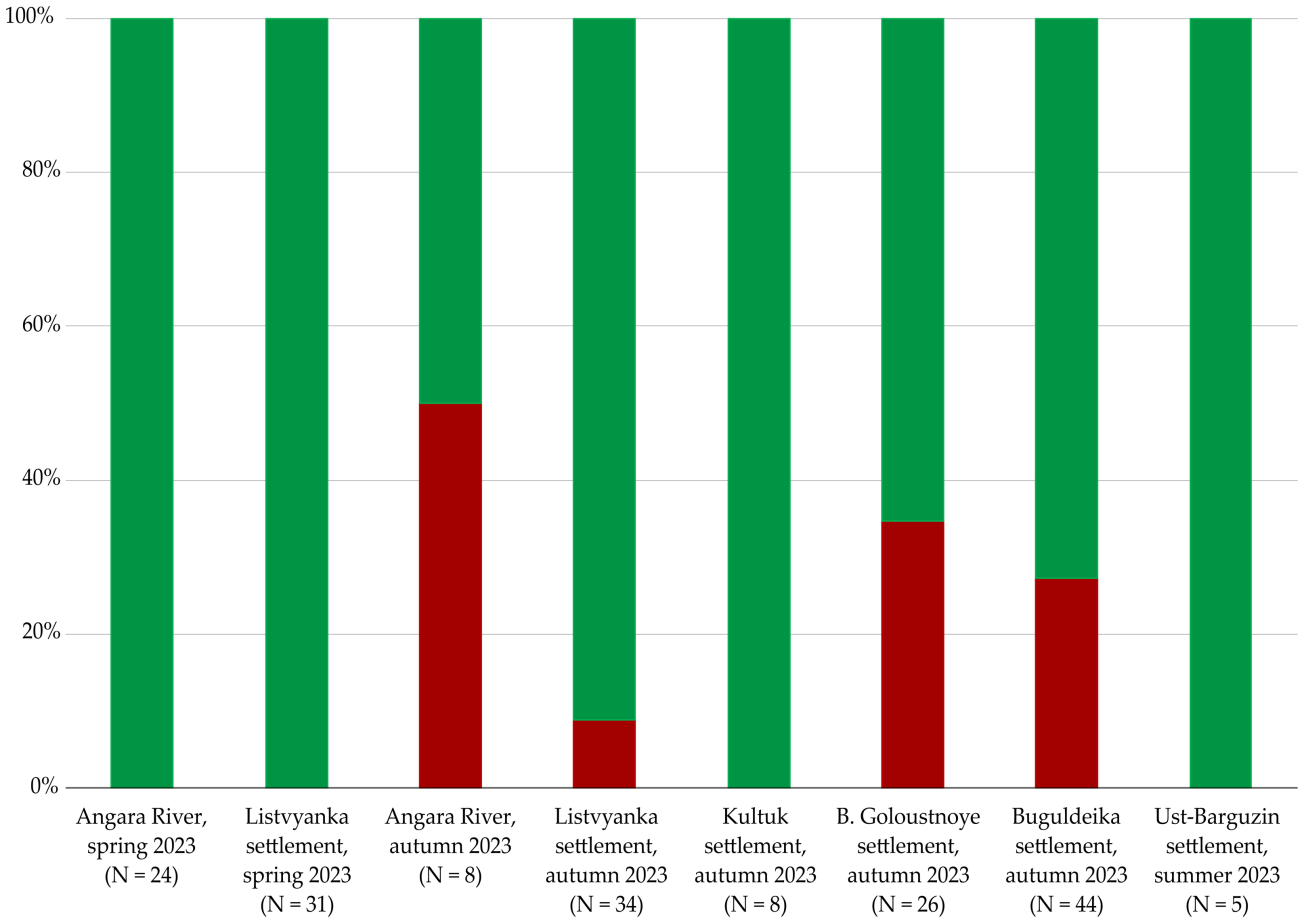
Evaluation of the presence of ibuprofen derivative in different populations of amphipod species *E. verrucosus*. Red—*E. verrucosus* contaminated with ibuprofen derivative; Green—clean samples in which ibuprofen derivative was not detected. Note: Contamination frequency is the percentage of analyzed amphipods in which the contaminant was detected.

Additionally, a compound structurally similar to ibuprofen was detected in several *E. verrucosus* specimens. While ibuprofen shows MRM transition 205.1 → 161.1, this compound exhibited a retention time shift of +0.2 min (Fig. 8C) with identical mass transitions. Figure 9 shows the mass spectra of the precursor ion and product ion of ibuprofen detected in Baikal amphipods. We hypothesize that this substance corresponds to the ibuprofen metabolite, since both substances have a similar fragmentation pattern and a similar retention time. The precursor ion has a charge mass after the breakdown of the molecule of 248.9, while the ion product has a mass of 160.9. The ibuprofen metabolite detected in amphipods is 3-carboxyibuprofen. 3-carboxyibuprofen was detected in *E. verrucosus* collected from the Angara River, Listvyanka, Bolshoye Goloustnoye and Buguldeika settlements (Fig. 10). The detection frequency of this derivative ranged from 10% to 50%. It was not detected in *E. verrucosus* from Kultuk and Ust-Barguzin settlements, nor in the deep-water amphipod *O. flavus*.

## Discussion

Pharmaceutical bioaccumulation in Lake Baikal’s ecosystem represents an urgent ecological concern. Initial studies confirmed trace drug residues in endemic amphipods, including acetylsalicylic acid, paracetamol, azithromycin, tetracyclines, amikacin, dimetridazole, metronidazole, and spiramycin. Earlier ibuprofen detection was reported by our studies, published by Telnova et al. (2024), who analyzed *E. verrucosus* from a Bolshoe Goloustnoe settl. in August 2020 and 2022. This monitoring revealed significant temporal variation: ibuprofen was detected in 70% of specimens in 2020 *versus* 27% in 2022 (Telnova et al., 2024). Our 2023 analysis revealed no direct ibuprofen contamination. Here we detected ibuprofen transformation product in 34% of *E. verrucosus* specimens. While ibuprofen metabolite screening was not conducted in 2020–2022, the declining parent compound concentration may reflect reduced post-pandemic pharmaceutical loading. These findings demonstrate that Baikal amphipods experience intermittent, but recurrent, pharmaceutical exposure with shifting contaminant profiles.

Quantitative analysis confirmed a significant inverse relationship between amphipod wet weight and accumulated ibuprofen concentrations. Maximum ibuprofen levels occurred in *E. verrucosus* from Buguldeika settl. Interspecific comparisons of *Eulimnogammarus* sp. and *Brandtia* sp. in the Angara River revealed differential bioaccumulation capacities. These patterns likely reflect morphological adaptations, particularly differences in integument permeability and exoskeleton composition affecting chemical uptake. Supporting this mechanism, Jakob et al. (2017) demonstrated reduced cadmium accumulation in larger *E. verrucosus versus* smaller *E. cyaneus*—attributable to allometric scaling of surface-area-to-volume ratios. Such size- and species-dependent accumulation principles appear conserved across pollutant classes, extending to pharmaceutical contaminants in Baikal amphipods.

Previous studies (Meyer et al., 2022) detected pharmaceuticals in Lake Baikal water including paracetamol (acetaminophen), paraxanthine, caffeine, cotinine, cimetidine, diphenhydramine, phenazone and sulphachloropyridazine at concentrations of 1–64 ng/L (equivalent to 0.001–0.064 ng/g). Contrastingly, our 2023 data reveal substantial bioaccumulation in endemic amphipods, with ibuprofen concentrations ranging from 4.19 to 1,151.32 ng/g—representing bioaccumulation factors (BAFs) of 65,500 to 1,151.320 relative to environmental concentrations (Chen et al., 2023). Conservative estimates indicate amphipods concentrate ibuprofen at 4,000–17,000 × environmental levels when compared to typical non-zero contaminant measurements (≥1 ng/L). This demonstrates exceptional biomagnification capacity within Baikal’s trophic web.

We propose that littoral *E. verrucosus* metabolizes ibuprofen, evidenced by a consistent 0.2-minute chromatographic retention time shift with identical mass fragmentation (MRM 205.1→161.1), indicating structural analogs. The discovered 3-carboxybuprofen is probably formed as a result of enzymatic or symbiotic biotransformation of amphipods. Notably, the metabolite was absent in *E. verrucosus* from Kultuk and Ust-Barguzin settlements.

The accumulation of active pharmaceutical ingredients like ibuprofen in Lake Baikal’s endemic amphipods originates from anthropogenic sources, entering the ecosystem *via* sewage discharge and groundwater infiltration—either as parent compounds or bioactive metabolites (Timoshkin et al., 2016). As a synthetic xenobiotic, ibuprofen lacks natural emission pathways. This contamination is exacerbated by Baikal’s status as a major recreational hub, where tourism and local usage drive pharmaceutical loading into nearshore environments.

This study identifies Listvyanka settlement as a principal ibuprofen contamination hotspot, where *E. verrucosus* amphipods exhibit dual exposure responses: bioaccumulation of parent compounds and metabolic transformation into derivatives. Anthropogenic loading likely stems from intensive tourism and inadequate wastewater treatment, compounded by seasonal dynamics. Quantitative analysis confirms significantly higher concentrations in spring-collected specimens *versus* autumn, potentially reflecting elevated pharmaceutical consumption during winter illness peaks (Kot-Wasik, Jakimska & Śliwka-Kaszyńska, 2016). Also, the water level of Lake Baikal is constantly experiencing seasonal and long-term changes, the inflow of water varies significantly throughout the year, and the volume of river water is unevenly distributed in certain parts of the lake (Rusinek et al., 2012). Thus, differences in concentrations can be explained depending on the sampling season.

Hydrographic processes critically govern ibuprofen distribution in Lake Baikal, where the counter-clockwise surface current (0.5–1.8 m/s) transports contaminants southward along the western shore from high-tourism zones (Maloe More and Olkhon Island) toward downstream settlements (Rusinek et al., 2012). This advective flow establishes a contamination gradient: Buguldeika—closest to northern sources (50 km from Olkhon) and directly within the current—exhibited peak concentrations (1,151 ng/g); Listvyanka (mid-transit, 85 km downstream) showed moderate levels (166 ng/g); while Kultuk, positioned beyond the Khamar-Daban coastal deflection zone and farthest from sources (120 km), showed no contamination. This spatial pattern correlates with Olkhon Island’s 300% tourism increase since 2015 (Aleksandrova et al., 2021), confirming current-mediated dispersal as the primary distribution mechanism for pharmaceutical pollutants in Baikal’s pelagic ecosystem.

As previously noted, pharmaceutical pollution in Lake Baikal’s waters is significantly driven by the absence of treatment facilities near populated areas and tourist hubs, improper disposal of expired medications, and seasonal disease outbreaks in humans. Pollutants enter the water through direct discharge of sewage from ships used in the tourism industry and other economic activities (Rusinek et al., 2012). There is virtually no system in place for collecting bilge and sewage water, and untreated effluent is discharged directly into the lake. There are certain threats from the inflow of pollutants *via* the lake’s tributaries, primarily the Selenga River. The catchment area of this river covers a significant area of territory where adequate control of untreated industrial and domestic wastewater is not always ensured (Rusinek et al., 2012). The selection of sampling locations is based on the above information.

Critically, no data currently exist on the effects of ibuprofen—or other pharmaceuticals—on amphipods or any endemic Baikal species. Nevertheless, synergistic interactions between pharmaceutical contamination and compounding stressors—including climate change, escalating cyanobacterial proliferation risks, the decline of Baikal sponges, and tourism pressures on sites like Olkhon Island—threaten to accelerate biodiversity loss and facilitate invasive species establishment in the near future (Timoshkin et al., 2016; Moore et al., 2009; Moore et al., 2019). Ongoing monitoring efforts have accumulated a substantial body of data, underscoring both the escalating environmental threat and the critical need for sustained surveillance and proactive conservation measures.

## Conclusions

This study establishes that endemic littoral and deep-water amphipods in Lake Baikal exhibit sustained ibuprofen contamination. We demonstrate for the first time that amphipods—potentially aided by symbiotic microorganisms—metabolize ibuprofen. Furthermore, Baikal amphipods bioaccumulate this pharmaceutical compound, with significantly higher concentrations observed in spring compared to autumn. Bioaccumulation capacity correlates positively with amphipod body mass, while several populations remain uncontaminated despite proximity to pollution sources. Collectively, these findings confirm that wild populations of endemic Baikal amphipods are chronically exposed to ibuprofen within their natural habitat.

##  Supplemental Information

10.7717/peerj.21008/supp-1 Supplemental Information 1 Categorical Data Designation of categorical data.

10.7717/peerj.21008/supp-2 Supplemental Information 2 Raw data on the manuscript

10.7717/peerj.21008/supp-3 Supplemental Information 3 Additional data related to the study

## Additional Information and Declarations

### Data Availability

The following information was supplied regarding data availability: The Raw data is available in the Supplemental Files.
