# Supplementary material for: Accumulation of ibuprofen in endemic amphipods of Lake Baikal
Source: PeerJ. 2026 Apr 9;14:e21008. doi: 10.7717/peerj.21008 (PMC13070318; doi:10.7717/peerj.21008)
Supplement: Supplemental Information 3 [file peerj-14-21008-s003.docx]

*Supplementary Materials*

**Accumulation of ibuprofen in endemic amphipods of Lake Baikal**

Tamara Yu. Telnova^1^, Maria M. Morgunova^1^, Sophie S. Shashkina^1^, Maria E. Dmitrieva^1^, Victoria N. Shelkovnikova^1^, Olga E. Lipatova^1^, Ekaterina V. Malygina^1^, Natalia A. Imidoeva^1^, Alexander Yu. Belyshenko^1^, Tatiana N. Vavilina^1^, Arcadii N. Matveev^2,3^, Evgenia A. Misharina^3^, Denis V. Axenov-Gribanov ^Corresp. 1,3^

^1^ Bioorganics Research and Educational Center, Irkutsk State University, Irkutsk, Russia

^2^ UNESCO Chair on Water Resources, Irkutsk State University, Irkutsk, Russia

^3^ Institute of biological sciences, Irkutsk State University, Irkutsk, Russia

Corresponding Author:

Denis V. Axenov-Gribanov^1,3^

Karl Marx Street, 1, Irkutsk 664003, Russia

Email address: [denis.axengri@gmail.com](mailto:denis.axengri@gmail.com)


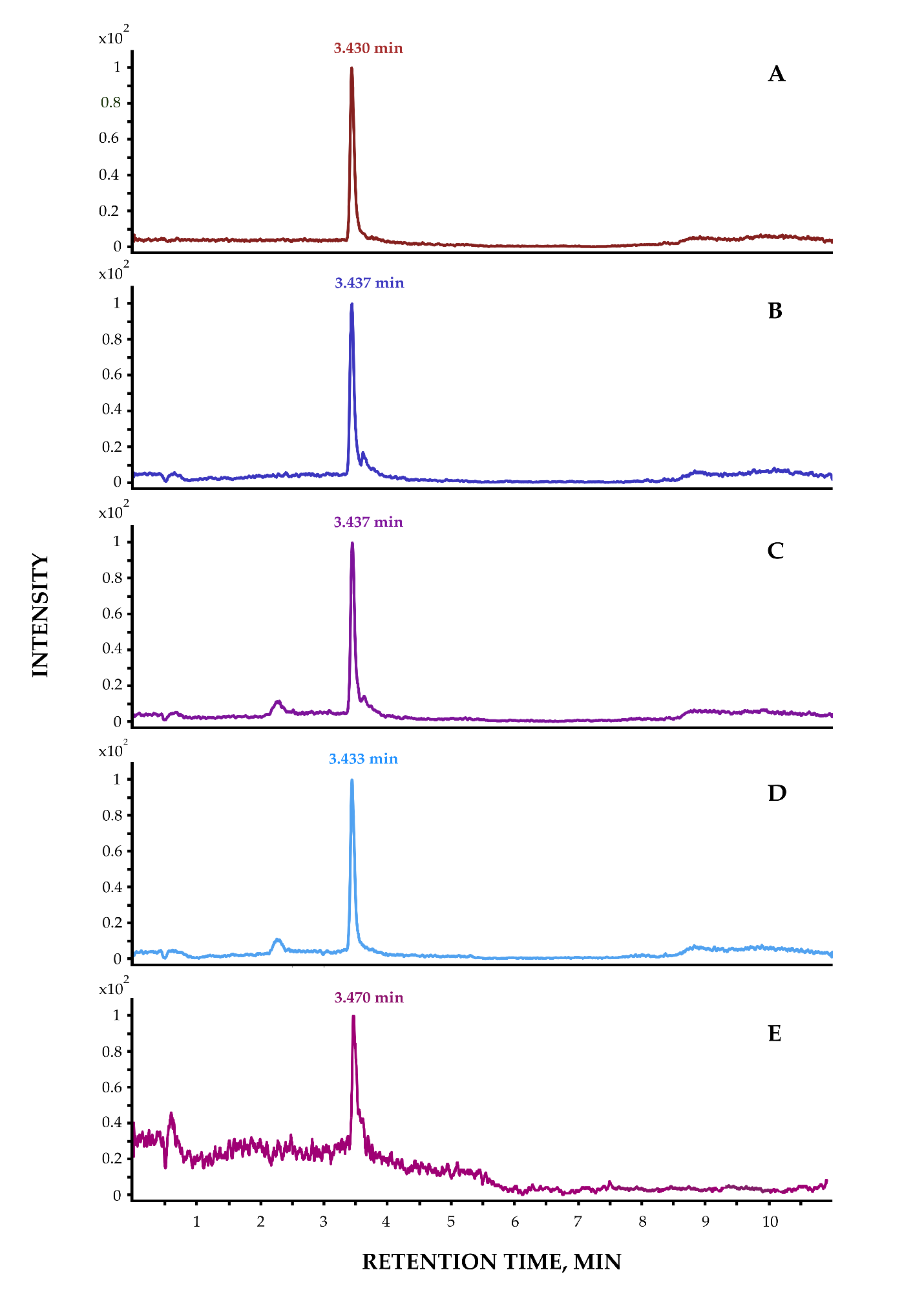
Figure S1. Typical chromatograms of qualitative analysis: a – analytical standard sample of ibuprofen (solution concentration 72 ng/ml); b – ibuprofen in amphipods of *Eulimnogammarus verrucosus* species; c – sample of amphipods of *E. verrucosus* species with the addition of an analytical standard sample of ibuprofen; d – ibuprofen in amphipods of *E. cyaneus* species; e – ibuprofen in amphipods of *Ommatogammarus flavus* species.

Table S1. Table of sampling sites for Baikal endemic amphipods.

| Seasonality | Location | Species |
| --- | --- | --- |
| Spring 2023 | Angara River | *Eulimnogammarus verrucosus*  *Eulimnogammarus* sp.  *Eulimnogammarus cyaneus*  *Brandtia* sp.  *Pallasea* sp. |
| Spring 2023 | Listvyanka settlement | *Eulimnogammarus verrucosus*  *Eulimnogammarus* sp.  *Brandtia* sp. |
| Spring 2023 | Buguldeika settlement | *Ommatogammarus flavus* |
| Autumn 2023 | Angara River | *Eulimnogammarus verrucosus* |
| Autumn 2023 | Listvyanka settlement | *Eulimnogammarus verrucosus* |
| Autumn 2023 | Buguldeika settlement | *Eulimnogammarus verrucosus* |
| Autumn 2023 | Kultuk settlement | *Eulimnogammarus verrucosus* |
| Autumn 2023 | Bolshoe Goloustnoye settlement | *Eulimnogammarus verrucosus* |
| Summer 2023 | Ust-Barguzin settlement | *Eulimnogammarus verrucosus* |
